# HMGB1-RAGE Axis Makes No Contribution to Cardiac Remodeling Induced by Pressure-Overload

**DOI:** 10.1371/journal.pone.0158514

**Published:** 2016-06-29

**Authors:** Hairuo Lin, Liang Shen, Xiajun Zhang, Jiahe Xie, Huixin Hao, Yingxue Zhang, Zhenhuan Chen, Hiroshi Yamamoto, Wangjun Liao, Jianping Bin, Shiping Cao, Xiaobo Huang, Yulin Liao

**Affiliations:** 1 State Key Laboratory of Organ Failure Research, Department of Cardiology, Nanfang Hospital, Southern Medical University, Guangzhou, 510515, China; 2 Department of Cardiology, first affiliated hospital of Jiaxing University, Jiaxing, Zhejiang, China; 3 Department of Oncology, Nanfang Hospital, Southern Medical University, Guangzhou, 510515, China; 4 Department of Biochemistry and Molecular Vascular Biology, Kanazawa University Graduate School of Medical Science, 13-1 Takara-machi, Kanazawa, 920-8640, Japan; Niigata University Graduate School of Medical and Dental Sciences, JAPAN

## Abstract

High-mobility group box1 (HMGB1) exerts effects on inflammation by binding to receptor for advanced glycation end products (RAGE) or Toll-like receptor 4. Considering that inflammation is involved in pressure overload-induced cardiac hypertrophy, we herein attempted to investigate whether HMGB1 plays a role in myocardial hypertrophy in RAGE knockout mice as well as in the growth and apoptosis of cardiomyocytes. The myocardial expression of RAGE was not significantly changed while TLR4 mRNA was upregulated in response to transverse aortic constriction (TAC) for 1 week. The myocardial expression of HMGB1 protein was markedly increased in TAC group when compared to the sham group. Heart weight to body weight ratio (HW/BW) and lung weight to body weight ratio (LW/BW) were evaluated in RAGE knockout (KO) and wild-type (WT) mice 1 week after TAC. Significant larger HW/BW and LW/BW ratios were found in TAC groups than the corresponding sham groups, but no significant difference was found between KO and WT TAC mice. Similar results were also found when TAC duration was extended to 4 weeks. Cultured neonatal rat cardiomyocytes were treated with different concentrations of recombinant HMGB1, then cell viability was determined using MTT and CCK8 assays and cell apoptosis was determined by Hoechst staining and TUNEL assay. The results came out that HMGB1 exerted no influence on viability or apoptosis of cardiomyocytes. Besides, the protein expression levels of Bax and Bcl2 in response to different concentrations of HMGB1 were similar. These findings indicate that HMGB1 neither exerts influence on cardiac remodeling by binding to RAGE nor induces apoptosis of cardiomyocytes under physiological condition.

## Introduction

High mobility group box 1 (HMGB1) is an evolutionarily conserved non-histone nucleoprotein with abundant expression in most mammalian cells [1,2]. Accumulating evidence indicates that its function now extends beyond the nucleus [3]. It can be actively secreted by innate immune cells [4–7] and passively released by damaged or necrosis cells [8] as a damage-associated molecular pattern. HMGB1 binds to receptor for advanced glycation end products (RAGE) [9] and members of Toll-like receptors (TLRs) such as TLR-2 and -4 [10,11], and participates in inflammation, immune response, apoptosis, regeneration and metastasis.

Apoptosis has been demonstrated to play a crucial role in the progress of heart failure, and it is known that the tumor suppressor gene Bax contributes to cardiomyocyte apoptosis [12]. Recent studies revealed that cells undergoing apoptosis release large amounts of HMGB1 [13]. However, whether HMGB1 promotes or inhibits apoptosis is still controversial. It is supported that endogenous HMGB1 enhances apoptosis during ischemia/reperfusion injury in heart [14], while it suppresses cellular apoptosis in osteosarcoma [15]. In addition, exogenous HMGB1 inhibits Lewis cell apoptosis [16]. These contrast findings suggest that the impact of HMGB1 on apoptosis maybe context-dependent.

Early studies have shown that hypertrophic stimulation increases translocation of HMGB1 from the nucleus to the cytoplasm, which is associated with DNA damage and cardiomyocyte hypertrophy, and exogenous HMGB1 stimulates cardiac regeneration [17,18]. Accumulated evidence shows that inflammation is involved in pressure overload-induced cardiac hypertrophy [19,20]. However, previous reports are controversial concerning the effect of HMGB1 on cardiomyocyte hypertrophy [17,21–24]. Myocardial pressure overload can induce systemic inflammation [19], while HMGB1 exerts proinflammatory activity by mainly binding to RAGE [25,26], therefore we hypothesized that deletion of RAGE would be beneficial for pressure overloaded heart.

In this study, we aimed to investigate the effect of HMGB1 on myocardial hypertrophy in RAGE deficient mice, and the role of recombinant HMGB1 (rHMGB1) on cell growth and apoptosis in cultured cardiomyocytes.

## Materials and Methods

All procedures were performed in accordance with our Institutional Guidelines for Animal Research and the investigation conformed to the Guide for the Care and Use of Laboratory Animals published by the US National Institutes of Health (NIH Publication No. 85–23, revised 1996). The study was approved by the ethics review board of Southern Medical University.

### Transverse aortic constriction (TAC) model

RAGE knockout (KO) mice were provided by Kanazawa University (Kanazawa, Japan), and heterozygotes were used to breed to generate homozygotes and wildtype (WT) mice. Adult mice (8–10 weeks old, male) were anaesthetized with a mixture of xylazine (5 mg/kg, intraperitoneally) and ketamine (100 mg/kg, intraperitoneally), and the adequacy of anesthesia was monitored by the disappearance of pedal withdrawal reflex. An endotracheal tube was inserted, and artificial respiration was supported by a respiratory machine. The pressure-overload model was created by TAC as described previously [27]. The mice were sacrificed by overdose of anesthesia with pentobarbital sodium (150 mg/kg, intraperitoneally) and cervical dislocation 1 or 4 weeks after TAC or sham operation. Heart weight to body weight ratio (HW/BW) and lung weight to BW ratio (LW/BW) were measured subsequently.

### Echocardiography

Transthoracic echocardiography was performed in mice 4 weeks after surgery using a Sequoia 512 system with a 17L-5 probe (Siemens, Germany). Mice were anesthetized with inhalational isoflurane at a concentration of 1.5%. Under M-mode, we measured the posterior wall thickness (LVPWd), end-diastolic diameter (LVEDd), and end-systolic diameter (LVESd). LV fractional shortening (FS) was calculated as follows: LVFS (%) = (LVEDd—LVESd) / LVEDd × 100.

### Invasive hemodynamic study

LV hemodynamics were evaluated in mice 4 weeks after surgery. Mice from each group were anesthetized with inhalational isoflurane at a concentration of 1.5% and were ventilated as mentioned above. A Millar catheter was inserted via the right carotid artery and carefully introduced into the LV to measure the systolic pressure (LVSP), end-diastolic pressure (LVEDP), and maximum and minimum rates of change of LV pressure (max d*p*/d*t* and min d*p*/d*t*, respectively).

### Histology

In mice with TAC or sham for 1 week, H&E staining and Azan-Masson were employed to evaluate myocardial hypertrophy and fibrosis, as described elsewhere [27,28].

### Polymerase chain reaction analysis (PCR)

Total RNA of homogenized murine whole heart was isolated using Total RNA Kit II (Omega) according to the protocol provided by the manufacturer. Quantitative real-time PCR was performed using a Quantitect SYBR Green RT-PCR kit (QIAGEN) and an Applied Biosystems 7500 system targeting the genes of RAGE, TLR4 and GAPDH.

### Cell culture

The neonatal rats were sacrificed by 2% isoflurane inhalation and cervical dislocation. The isolation and culture of neonatal rat ventricular cardiomyocytes (NRVCs) were performed as described previously [29,30]. NRVCs were cultured for 1 day and treated with various concentrations of HMGB1 (Sigma-Aldrich Co.) and/or anti-RAGE neutralizing antibody (Merck Millipore Co).

### Western blot analysis

Proteins were extracted from the cultured cardiomyocytes or the murine heart. Immunoblotting was performed using antibodies against HMGB1 (Cell Signaling Technology), cytosolic Bax (cyto-Bax), total-Bax, Erk and phosphor-Erk (Cell Signaling Technology) and Bcl2 (Santa Cruz Biotechnology). Blotting of β-actin (Santa Cruz Biotechnology) was used as a loading control. Immunoreactive bands were visualized with Odyssey^®^ Infrared Imaging System (LI-COR^®^ Bioscience) and quantified by densitometry with Image J software (NIH).

### Measurements of cell viability and apoptosis

Cell viability was determined using the MTT (Sigma-Aldrich Co) kit and CCK8 kit (Dojindo, Tokyo, Japan) according to our previous report [28,29]. Apoptosis in NRVCs was detected by Hoechst staining and TUNEL assay as we described previously [28,31]; cells were fixed with 4% (w/v) formaldehyde in phosphate buffered saline for 10 min, and then stained with Hoechst 33258 (10 mg/l) (Beyotime, China) for 15 min, and the results were observed under fluorescence microscopy. Apoptotic cells were indicated by bright white nuclei.

### Statistical analysis

Quantitative data were expressed as mean ± SEM (standard error of mean). Normal distribution test was performed to determine parameter or non-parameter tests should be used. Comparison between two experimental groups was based on a two-tailed t-test, while comparisons of parameters among ≥3 groups were analyzed by ANOVA followed by Bonferroni’s correction for post hoc multiple comparisons. In all analyses, differences were considered statistically significant at a value of *P* < 0.05.

## Results

### RAGE deletion did not attenuate cardiac hypertrophy

**Fig 1:**
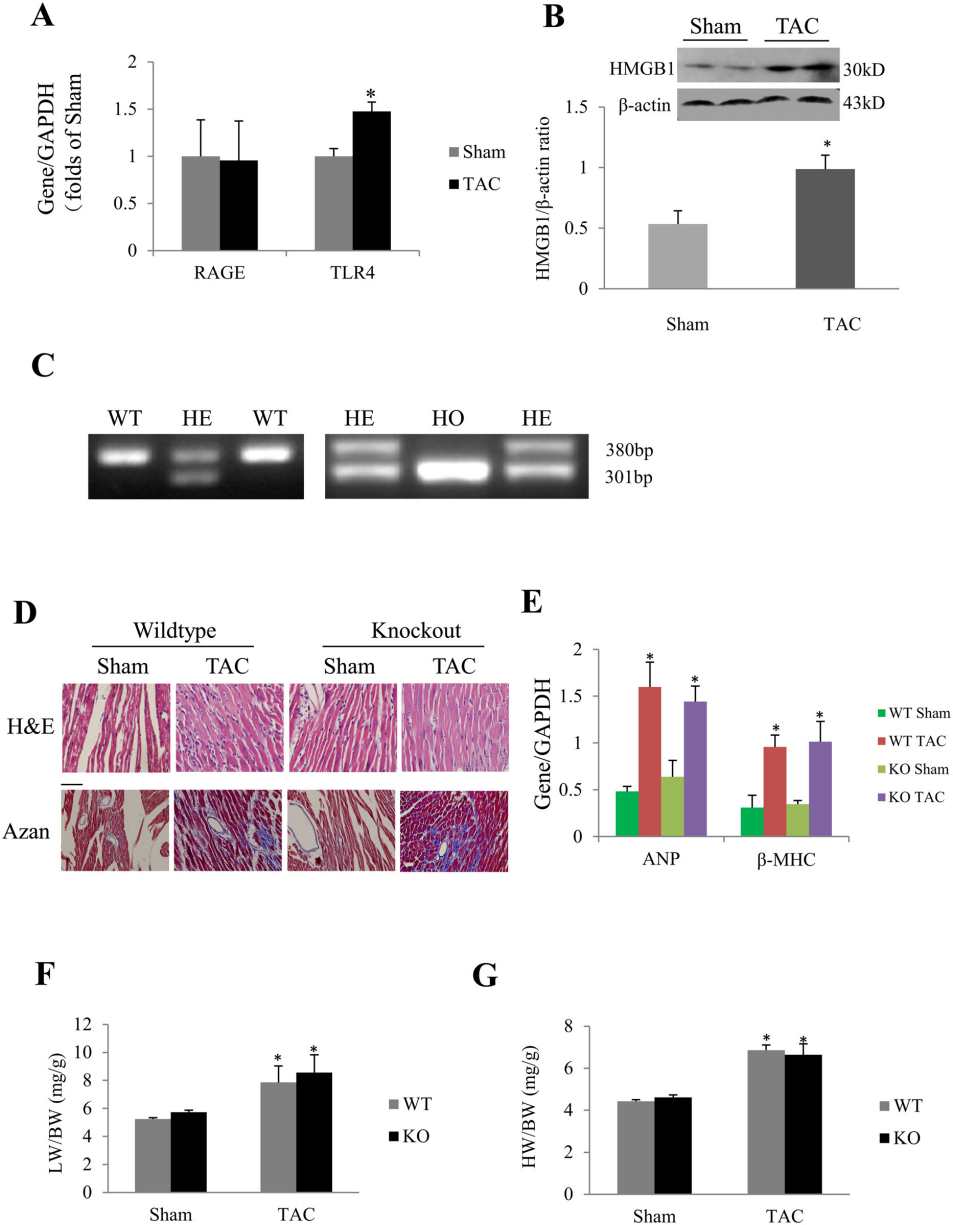
Effect of RAGE deletion on cardiac hypertrophy induced by TAC for 1 week. (A) Results of Real-time PCR for gene expression of receptor for advanced glycation end products (RAGE) and Toll like receptor 4 (TLR4) in mice subjected to sham or transverse aortic constriction (TAC). (B) Western blot of high-mobility group box1 (HMGB1) expression in response to sham or TAC. *P < 0.05 compared with sham, n = 5 in each group for panel A and B. (C) Genotyping results. RAGE −/− (homozygote, HO) and RAGE +/+ (wildtype, WT) mice for experiments were generated by mating RAGE + / − (heterozygote, HE) mice with each other. (D) H&E and Azan staining to evaluate cardiomyocyte hypertrophy and myocardial fibrosis, respectively. Scale bar = 50 μm. (F) Lung weight/body weight (LW/BW) ratio was similar in WT and KO (RAGE knockout) TAC mice. (G) Heart weight/body weight (HW/BW) ratio in response to sham or TAC. *P < 0.01 compared with the corresponding sham group. Results in histograms are mean ± SEM, n = 10 in WT sham and WT TAC groups, and n = 7 and 6 in KO sham and KO TAC group, respectively.

One week after TAC, the myocardial expression of RAGE detected by quantitative real-time PCR was not significantly changed, while the TLR4 mRNA was increased (Fig 1A). In addition, the myocardial expression of HMGB1 protein was markedly increased in response to TAC (Fig 1B).

Heterozygous RAGE mice were bred at our animal facility to produce RAGE −/−, RAGE −/+ and RAGE +/+ offspring (Fig 1C). The mortality after TAC for 1 week was similar between WT and KO mice (5/15 in WT group, 4/10 in KO group). No significant difference was found between WT TAC group and KO TAC group on histological findings (H&E stain and Masson stain. Fig 1D, also see Figs A and B in S1 File) and hypertrophic markers of ANP and β-MHC (Fig 1E). Lung weight to body weight ratio (LW/BW) and heart weight to body weight ratio (HW/BW) were significantly larger in RAGE KO and WT mice 1 week after TAC compared with the corresponding sham groups, but no significant difference was found between KO TAC and WT TAC mice (Fig 1F and 1G).

### RAGE deletion did not improve TAC-induced heart failure

**Fig 2:**
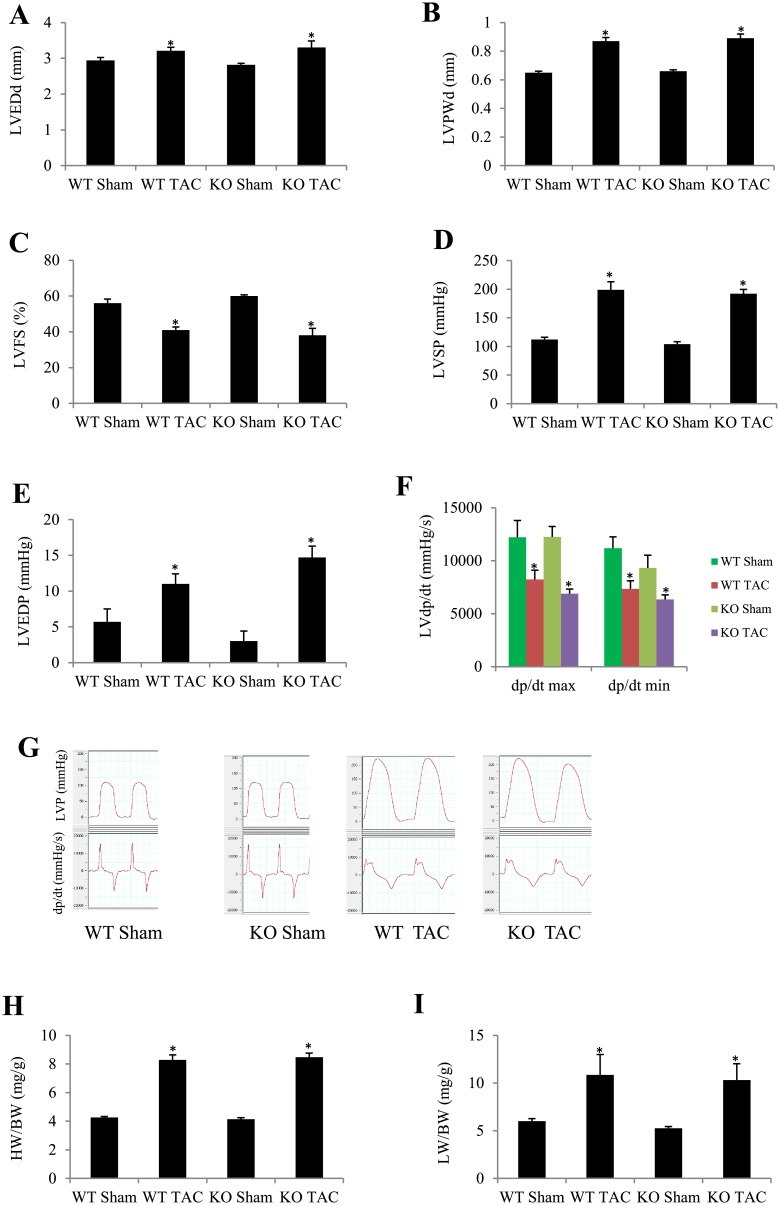
Effect of RAGE deletion on heart failure induced by TAC for 4 weeks evaluated by echocardiography, invasive hemodynamic measurements and morphological analysis. (A) Echocardiographic left ventricular end-diastolic diameter (LVEDd). (B) Echocardiographic LV posterior wall thickness at diastole (LVPWd). (C) Echocardiographic LV fractional shortening (LVFS). (D) LV systolic pressure (LVSP). (E) LV end-diastolic pressure (LVEDP). (F) LV dp/dt max and min. (G) Examples of LV pressure and dp/dt curves recoding. (H) Heart weight to body weight ratio (HW/BW). (I) Lung weight to body weight ratio (LW/BW). Results are mean ± SEM, n = 6–8 in each group, *P < 0.05 vs. the corresponding sham group.

We further investigated the influence of RAGE deletion on heart failure by extending TAC duration to 4 weeks. The survival rate following TAC was similar between KO (7/11) and WT mice (7/10). Echocardiographic measurements showed that LVEDd and LVPWd were significantly larger, while LVFS was smaller in TAC groups than in sham groups, but no significant difference was found between WT TAC group and KO TAC group (Fig 2A–2C). Results of LV hemodynamic are shown in Fig 2D–2G. LVSP (Fig 2D and 2G) and LVEDP (Fig 2E and 2G) were higher and ±dp/dt max (Fig 2F and 2G) was lower in TAC groups than their corresponding sham groups, but no significant difference was found between WT TAC group and KO TAC group (Fig 2D–2G). Similarly, HW/BW and LW/BW were significantly larger in RAGE KO and WT mice 4 weeks after TAC compared with the corresponding sham groups, but no significant difference was found between KO and WT TAC mice (Fig 2H and 2I).

### HMGB1 exerted no effect on cardiomyocyte apoptosis

**Fig 3:**
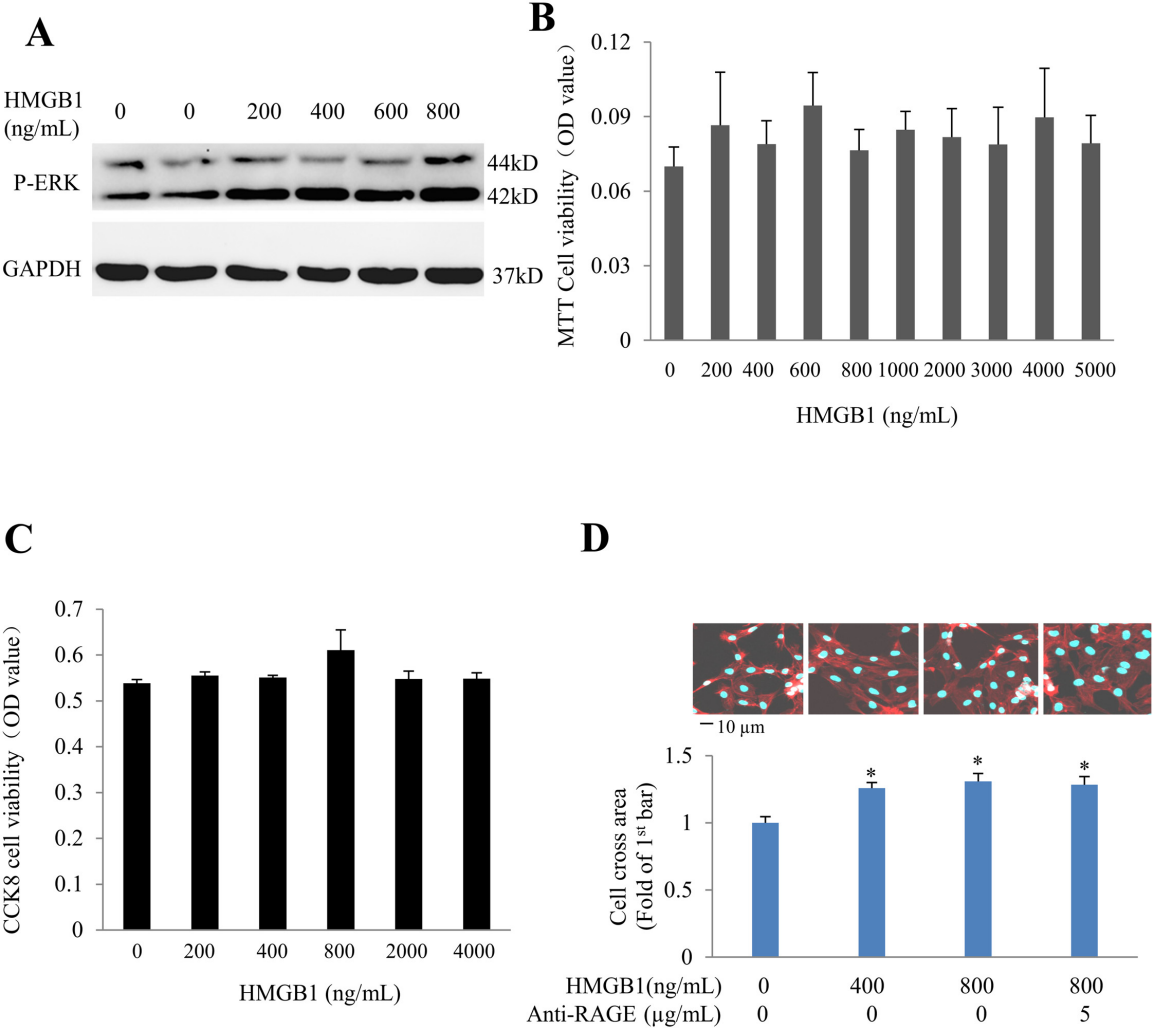
Effect of rHMGB1 on cell viability. Neonatal rat cardiomyocytes were exposed to recombinant HMGB1 (rHMGB1). (A) Effect of rHMGB1 on p-ERK1/2. (B) Cell viability was detected with MTT assay. (C) Cell proliferation detected with CCK8 assay. (D) Cell surface area in response to rHMGB1 in the absence/presence of anti-RAGE neutralizing antibody. Results are means ± SEM. n = 6 in each dose group in panel A-C, n = 50 cells in each group and *P <0.05 vs. 1^st^ bar in panel D.

**Fig 4:**
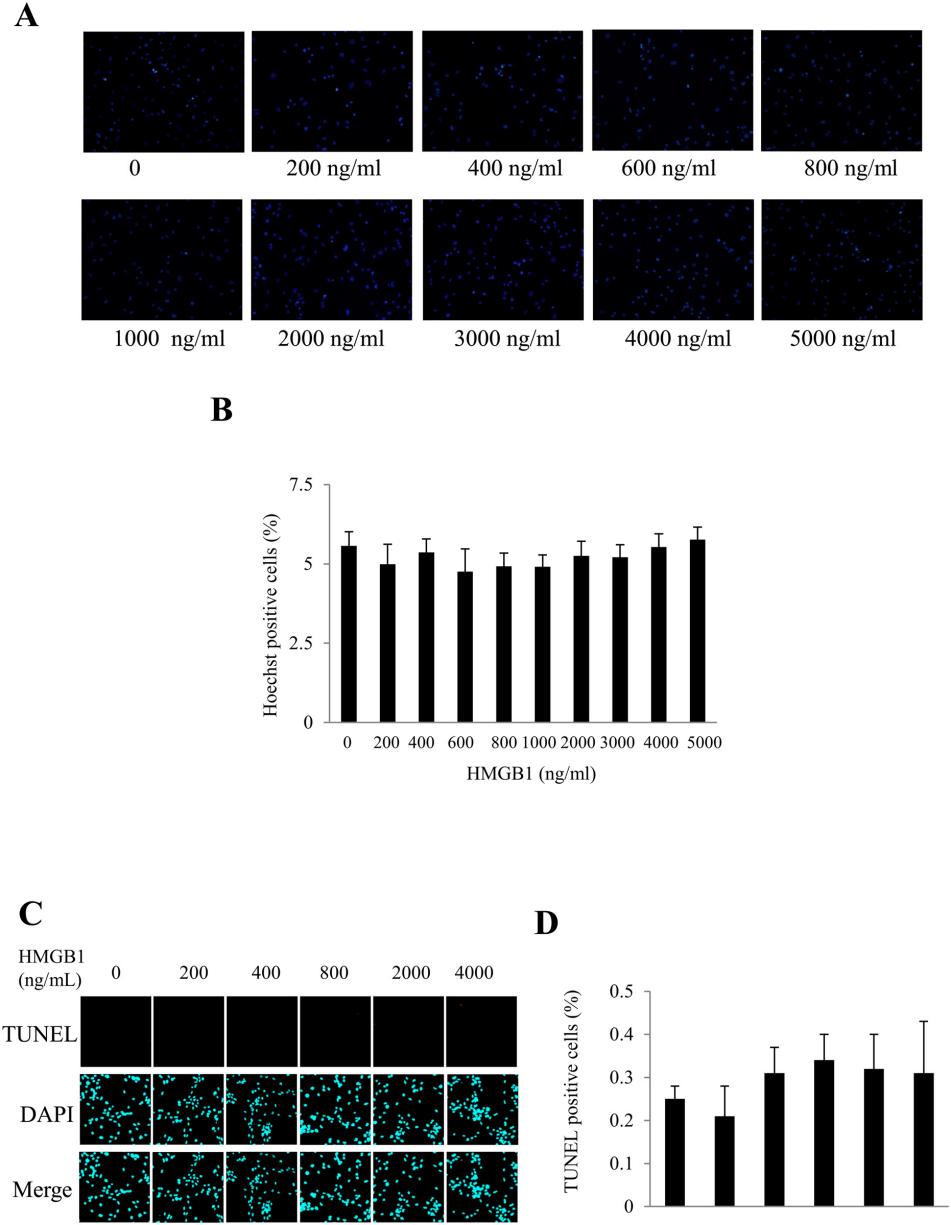
Effect of rHMGB1 on apoptosis of cardiomyocytes. Neonatal rat **c**ardiomyocytes were stimulated with different concentrations of rHMGB1 for 24h. (A) Hoechst staining assay of cardiomyocyte apoptosis. Scale bar, 50 μm. (B) Quantitative analysis of Hoechst-staining positive cells. (C) Examples of TUNEL staining pictures. (D) Quantitative analysis of TUNEL-assay positive cells. Results in histograms are mean ± SEM. Each experiment was repeated at least three times.

In order to explore the role of HMGB1 on cardiomyocyte apoptosis, neonatal rat **c**ardiomyocytes were exposed to different concentrations of recombinant HMGB1 (rHMGB1) for 24h. We confirmed that rHMGB1 can induce phosphorylation of ERK1/2 (Fig 3A). In cultured NRVCs, stimulation with rHMGB1 didn’t reduce the cell viability measured by either MTT assay or CCK8 assay (Fig 3B and 3C), but increased cardiomyocyte hypertrophy significantly which was not antagonized by co-treatment with anti-RAGE neutralizing antibody (Fig 3D), indicating that HMGB1-induced cardiomyocyte hypertrophy is RAGE-independent. rHMGB1 didn’t increase the apoptosis of cardiomyocytes determined by Hoechst staining or TUNEL staining (Fig 4A–4D).

### HMGB1 exerted no effect on the protein expression of Bax and Bcl2

**Fig 5:**
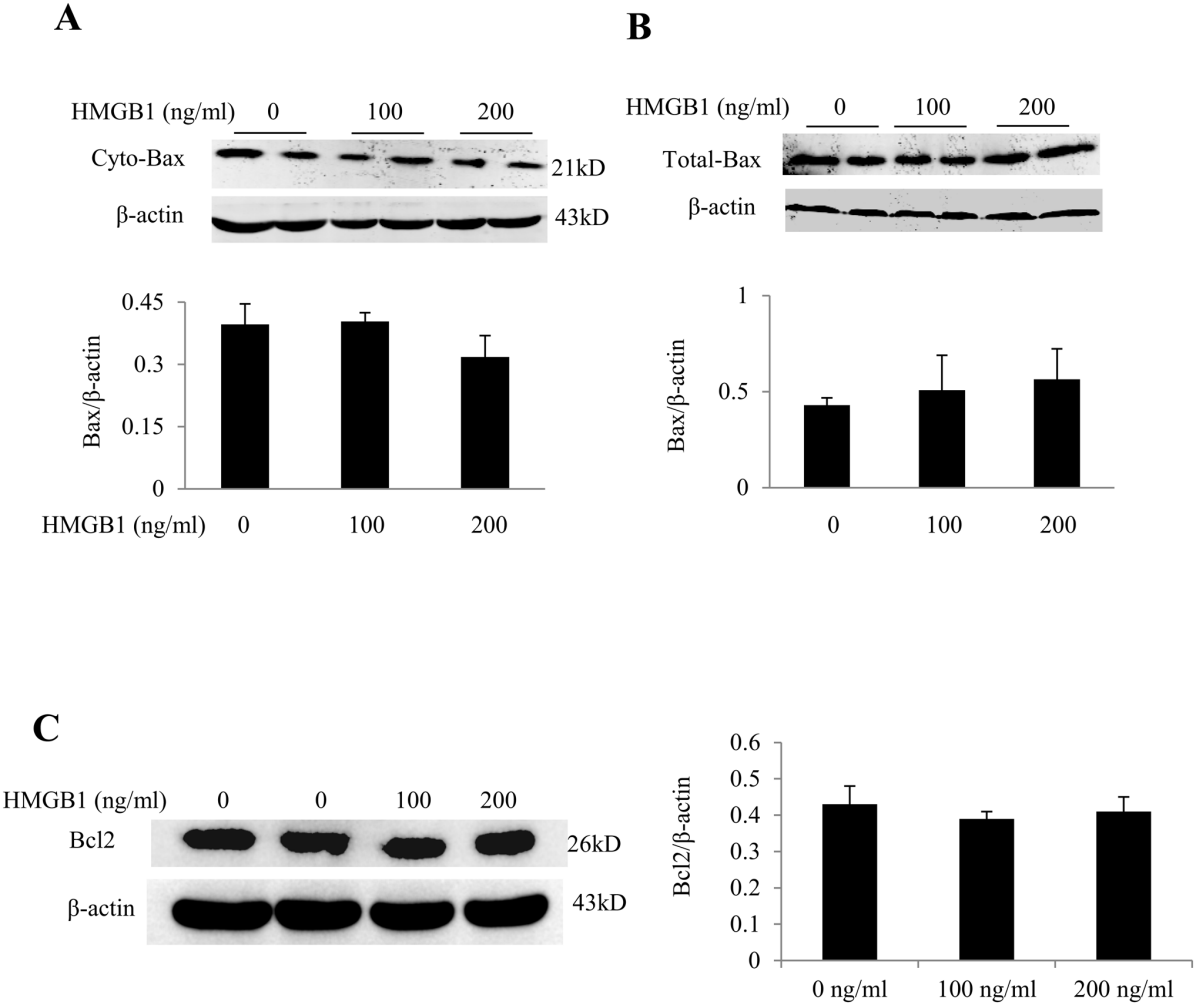
rHMGB1 exerted no effect on protein expression of Bax and Bcl2 in neonatal rat cardiomyocytes. (A) Western blot analysis of cytosolic Bax (cyto-Bax) in the presence of rHMGB1. (B)Total cellular protein expression of Bax. (C) Total cellular protein expression of Bcl2. β-actin served as a loading control. Results are means ± SEM. Each experiment was repeated three times.

To determine the effect of HMGB1 on mitochondrial translocation of Bax, we evaluated the expression of cytosolic and total cellular Bax protein. We noted that HMGB1 exerted no effect on cytosolic (Fig 5A) or total cellular Bax protein expression (Fig 5B). Similarly, no significant effect of rHMGB1 on Bcl2 protein expression was noted (Fig 5C). These findings indicated that HMGB1 does not increase mitochondrial translocation of Bax or cardiomyocyte apoptosis.

## Discussion

A recent report demonstrated that myocardial expression of HMGB1 protein and its translocation from nucleus to both cytoplasm and intercellular space were increased in mice with TAC, and exogenous HMGB1 aggravated TAC-induced cardiac hypertrophy [32], but it is unclear whether HMGB1-RAGE axis is involved in cardiac hypertrophy. In this study, we also found that HMGB1 was significantly increased in response to pressure overload. If the endogenous HMGB1 enhances cardiac hypertrophy by binding to RAGE, TAC-induced hypertrophy should be attenuated in RAGE KO mice. However, in this study, we demonstrated that there was no significant difference in HW/BW between the RAGE WT TAC and KO TAC mice, implicating that endogenous HMGB1-RAGE doesn’t contribute to cardiac hypertrophy.

HMGB1 has been shown to induce calcineurin-mediated cell hypertrophy in neonatal rat cardiomyocytes [21], while upregulated-HMGB1 under pressure overload promoted cardiac hypertrophy and heart failure [32]. In addition, inhibiting HMGB1 release or promoting nuclear translocation of HMGB1 was also reported to attenuate cardiac hypertrophy [17,22,24]. These studies indicate that HMGB1 has a cellular-location-dependent influence on cardiomyocyte hypertrophy. HMGB1 exerts its biological activity through binding to its receptor RAGE or TLR4. However it is unclear which receptor is involved in HMGB1-related cardiac hypertrophy. Previous studies support that activation of TLR4 contributes to cardiac hypertrophy, while genetic or pharmacological inhibition of TLR4 attenuates myocardial hypertrophy [33–35]. Evidence is limited concerning the role of RAGE in cardiac hypertrophy. Yan et al reported that S100/calgranulin is associated with the development of cardiac hypertrophy in a RAGE-dependent manner in a murine model of chronic kidney disease [36]. It is unknown whether RAGE deficiency would influence cardiac hypertrophy that is not induced by diabetic mellitus or chronic kidney disease. Although our findings in this study can’t answer whether HMGB1-RAGE is involved in any types of cardiac hypertrophy, we can conclude that HMGB1-RAGE is not involved in pressure overload-induced cardiac hypertrophy.

We have characterized the murine pressure-overload model in a time course and confirmed that 1–2 weeks TAC induces myocardial hypertrophy without heart failure and 4 weeks or longer TAC induces chronic heart failure [27,37]. In this study, we noted that no significant influence of RAGE deletion on cardiac remodeling was noted in mice with TAC for 4 weeks. HMGB1 exerts proinflammatory activity by mainly binding to RAGE. Recent report demonstrated that HMGB1-RAGE axis is involved in the pathogenesis of inflammatory cardiomyopathy in mice with troponin I immunization [25], while it is well known that inflammation is also closely associated with heart failure [38]. Our results suggest that HMGB1-RAGE axis is a redundant inflammatory factor in pressure-overload induced heart failure.

It is believed that HMGB1 is involved in apoptosis, but the exact relationship between HMGB1 and apoptosis is still controversial. In this study, we demonstrated that exogenous HMGB1 neither decreased the cell viability of NRVCs nor increased the Hoechst staining positive nuclei, despite the concentration was varied from 0 to 5000 ng/ml. As we know, the tumor-suppressor gene Bax could contribute to cardiomyocyte apoptosis, which would translocate into mitochondria during apoptosis. In this study, we found that HMGB1 stimulation in different concentrations did not change the expression of total or cytosolic Bax, which indicates that HMGB1 doesn’t increase mitochondrial translocation of Bax or cardiomyocyte apoptosis as well. Many studies have explored the effects of HMGB1 on cardiomyocyte apoptosis. Ding et al found that HMGB1-enhanced cardiomyocyte apoptosis contributed to myocardial ischemia/reperfusion injury [39], and Wang et al reported that HMGB1 promoted hyperglycemia-induced cardiomyocyte apoptosis [40]. In contrast, Narumi et al showed that intracellular HMGB1 attenuated doxorubicin-induced cardiomyocyte apoptosis [41]. So it seems hard to conclude that HMGB1 promotes or inhibits apoptosis under pathological stress. In this study, we used recombinant HMGB1 in different concentrations to stimulate NRVCs directly in the absence of pathological stimulations, and definitely demonstrated the direct effect of HMGB1 on cardiomyocytes. However, we did not investigate the effect of HMGB1 on cardiomyocyte apoptosis under pathological states, which is the limitation of this study which should be explored in further studies.

In conclusion, HMGB1 is up-regulated in pressure overload-induced cardiac hypertrophy and exerts no effect on RAGE-mediated cardiac hypertrophy, and HMGB1 doesn’t influence apoptosis of cardiomyocytes under physiological state.

## Supporting Information

S1 File Fig A. Representative pictures of H&E staining. Cardiomyocyte hypertrophy in mice deficiency of receptor for advanced glycation end products (RAGE) and their wild type (WT) littermates in response to pressure overload for 1 week. KO: RAGE knockout; TAC: transverse aortic constriction. Fig B. Representative pictures of Azan-Mason staining. Myocardial fibrosis in mice deficiency of receptor for advanced glycation end products (RAGE) and their wild type (WT) littermates in response to pressure overload for 1 week. KO: RAGE knockout; TAC: transverse aortic constriction.
(PDF)
