# Correction: HMGB1-RAGE Axis Makes No Contribution to Cardiac Remodeling Induced by Pressure-Overload

**DOI:** 10.1371/journal.pone.0167872

**Published:** 2016-12-02

**Authors:** The PLOS ONE Staff

The following information is missing from the Funding section: This study was supported by grants from the National Natural Science Foundation of China (31271513) to Professor Yulin Liao; the Provincial Natural Science Foundation of Guangdong (2014A030313342) to Professor Yulin Liao; the Provincial Natural Science Foundation of Guangdong (2015A030313301) to Dr. Xiaobo Huang; President Foundation of Nanfang Hospital, Southern Medical University (2014B019) to Dr. Xiaobo Huang; the Provincial Nature Foundation of Guangdong (2015A030313298) to Shiping Cao. The publisher apologizes for this error.
