# Supplementary material for: HMGB1-RAGE Axis Makes No Contribution to Cardiac Remodeling Induced by Pressure-Overload
Source: PLoS One. 2016 Jun 29;11(6):e0158514. doi: 10.1371/journal.pone.0158514 (PMC4927190; doi:10.1371/journal.pone.0158514)
Supplement: S1 File — Fig A. Representative pictures of H&E staining. Cardiomyocyte hypertrophy in mice deficiency of receptor for advanced glycation end products (RAGE) and their wild type (WT) littermates in response to pressure overload for 1 week. KO: RAGE knockout; TAC: transverse aortic constriction. Fig B. Representative pictures of Azan-Mason staining. Myocardial fibrosis in mice deficiency of receptor for advanced glycation end products (RAGE) and their wild type (WT) littermates in response to pressure overload for 1 week. KO: RAGE knockout; TAC: transverse aortic constriction. (PDF) [file pone.0158514.s001.pdf]

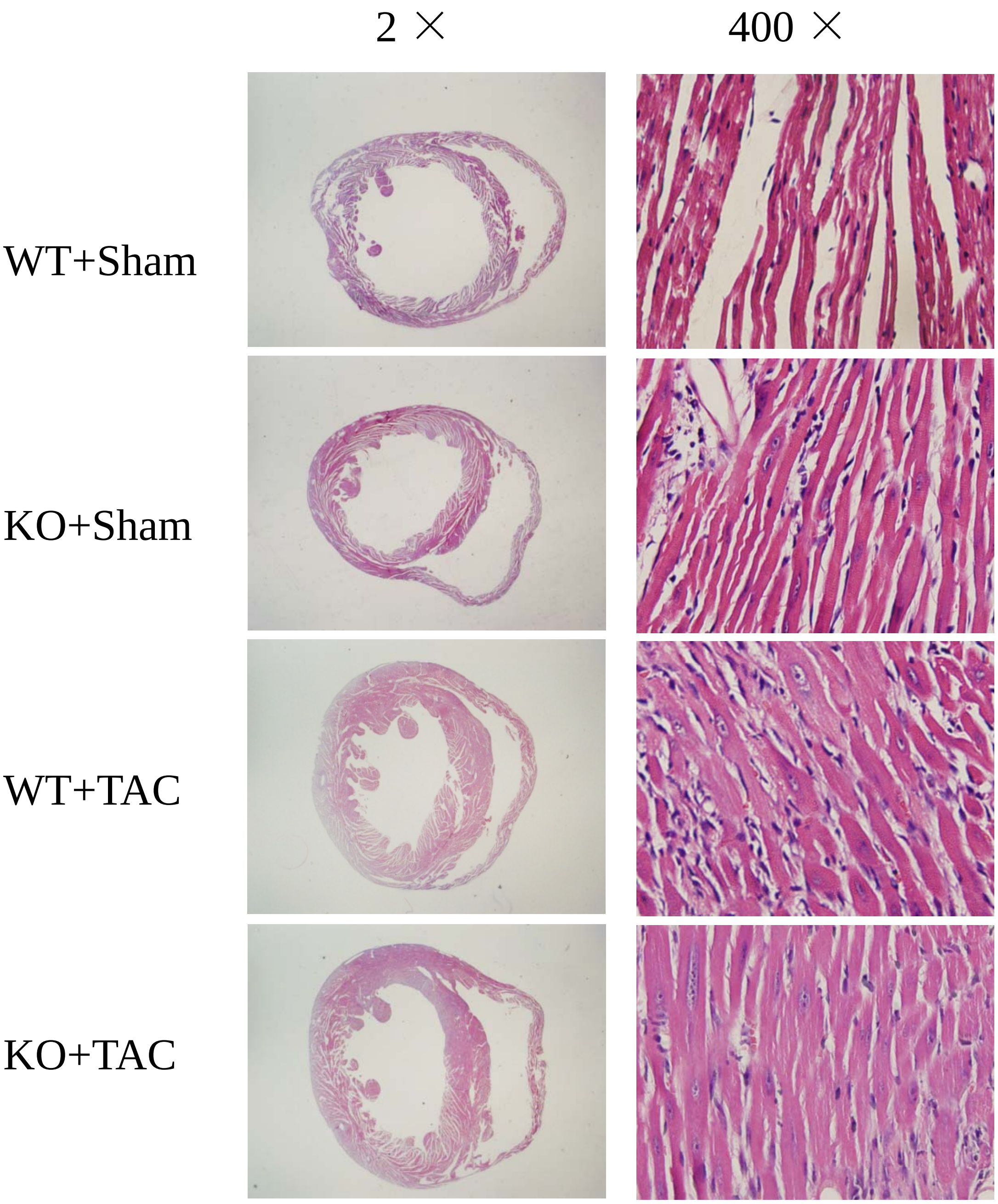

Fig. A Representative pictures of H&E staining. Cardiomyocyte hypertrophy in mice deficiency of receptor for advanced glycation end products (RAGE) and their wild type (WT) littermates in response to pressure overload for 1 week. KO: RAGE knockout; TAC: transverse aortic constriction.

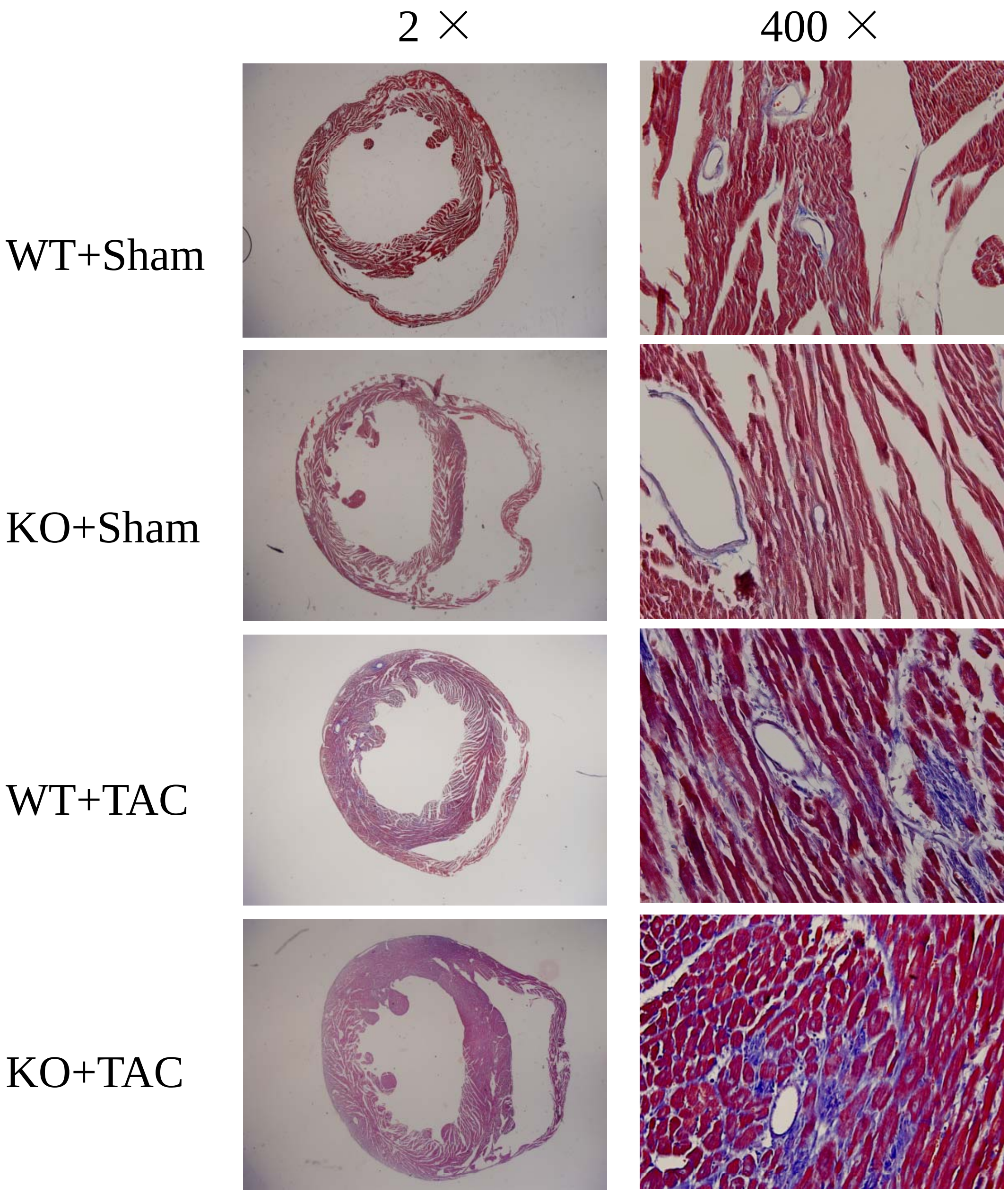

Fig B Representative pictures of Azan-Mason staining. Myocardial fibrosis in mice deficiency of receptor for advanced glycation end products (RAGE) and their wild type (WT) littermates in response to pressure overload for 1 week. KO: RAGE knockout; TAC: transverse aortic constriction.
